# Stimulatory Effect of 5-Hydroxytryptamine (5-HT) on Rat Capsaicin-Sensitive Lung Vagal Sensory Neurons via Activation of 5-HT_3_ Receptors

**DOI:** 10.3389/fphys.2019.00642

**Published:** 2019-05-28

**Authors:** Chun-Chun Hsu, Ting Ruan, Lu-Yuan Lee, You Shuei Lin

**Affiliations:** ^1^School of Respiratory Therapy, College of Medicine, Taipei Medical University, Taipei, Taiwan; ^2^Division of Pulmonary Medicine, Department of Internal Medicine, Taipei Medical University Hospital, Taipei, Taiwan; ^3^Department of Physiology, University of Kentucky Chandler Medical Center, Lexington, KY, United States; ^4^School of Medicine, Fu Jen Catholic University, New Taipei City, Taiwan; ^5^Department of Physiology, School of Medicine, College of Medicine, Taipei Medical University, Taipei, Taiwan

**Keywords:** airway, C-fiber, inflammation, serotonin, reflex, afferent

## Abstract

5-hydroxytryptamine (5-HT) is an inflammatory mediator known to be released in lung. Capsaicin-sensitive lung vagal (CSLV) afferents function as a primary sensor for detecting chemical stimuli and produce consequent reflexes during lung inflammation. To characterize the effect of 5-HT on CSLV afferents, responses of cardiorespiratory reflexes and single-unit C-fiber afferents to right-atrial injections of 5-HT were investigated in anesthetized Sprague-Dawley rats. Bolus injection of 5-HT (8 μg/kg) caused an immediate augmented breath and apnea, accompanied by hypotension and bradycardia. These initial responses were then followed by a brief pressor response and a more sustained depressor response. After a perineural treatment of both cervical vagi with capsaicin to block the conduction of C fibers, 5-HT still triggered the augmented breath, but no longer evoked the apnea, bradycardia and hypotension, indicating an involvement of C-fiber activation. The remaining augmented breath induced by 5-HT after perineural capsaicin treatment was totally eliminated by vagotomy. To further study the effect of 5-HT on CSLV afferents, activities arising from these afferents were determined using the single-fiber recording technique. Right-atrial injection of 5-HT evoked an intense discharge in CSLV afferents in a dose-dependent manner. The highest dose of 5-HT (16 μg/kg) activated 79% (19/24) of CSLV afferents which were also sensitive to capsaicin (0.8 μg/kg). The pretreatment of tropisetron, a selective antagonist of the 5-HT_3_ receptor, completely blocked CSLV-afferents stimulation induced by 5-HT but did not affect that by capsaicin. Furthermore, a similar afferent response of CSLV afferents was mimicked by phenylbiguanide, a selective agonist of the 5-HT_3_ receptor. In isolated rat lung vagal C neurons, 5-HT induced intense calcium transients in a dose-dependent manner. The highest concentration (3 μM) of 5-HT activated 67% (18/27) of the CSLV neurons. The 5-HT-induced response was totally abolished by pretreatment of tropisetron. In conclusion, 5-HT exerts an intense stimulatory effect on lung C-fiber terminals mediated through an activation of the 5-HT_3_ receptor, which may contribute to the airway hypersensitivity under lung inflammation.

## Introduction

5-hydroxytryptamine (5-HT), a monoamine, is known to contribute to the nociception of tissue injury by stimulation of nociceptive sensory nerves via activation of 5-HT_3_ receptors (Zeitz et al., 2002). In the lungs, 5-HT is released mainly by platelets (McNicol and Israels, 1999) and is known to contribute to the pathogenesis of various adverse pulmonary responses such as bronchoconstriction (Buckner et al., 1993; Martin et al., 1994) and airway edema (Sumita et al., 1989; Lima et al., 2007).

Pulmonary sensory nerves are believed to be involved in many of these pulmonary responses induced by 5-HT (Dutta and Deshpande, 2010). The majority of sensory nerve fibers innervating the respiratory tract are capsaicin-sensitive lung vagal (CSLV) afferents (Agostoni et al., 1957), the nociceptive sensory nerve endings. These fibers are highly chemosensitive and can be activated by several endogenous mediators whose releases are elevated during lung inflammation (Carr and Undem, 2003; Hsu et al., 2013). Activation of the CSLV afferents elicits diffusive pulmonary responses such as cough, bronchoconstriction, and edema of airway mucosa in various species including humans (Lee and Pisarri, 2001; Carr and Undem, 2003).

Stimulation of CSLV afferents has been suggested to contribute to the 5-HT-induced airway reflexes, based on the fact that 5-HT activates CSLV neurons (Hong et al., 1997; Widdicombe, 2003; Chuaychoo et al., 2005) via activation 5-HT_3_ receptor (Potenzieri et al., 2012). However, this hypothesis has been challenged by the finding that phenylbiguanide (PBG, a 5-HT_3_ agonist) triggers apneic reflex and activates vagal afferents, but these PBG sensitive afferents have no response to capsaicin, a potent and selective CSLV activators (Dutta and Deshpande, 2010). In addition, 5-HT has also been shown to evoke bronchoconstriction a direct action on non-neuronal tissue such as airway smooth muscles (Szarek et al., 1995), which may indirectly activate vagal mechanoreceptors such as rapidly adapting receptors (RARs) (Widdicombe, 2003). Whether CSLV afferents are responsible for the 5-HT-induced pulmonary reflexes needs to be further determined.

In light of the existing information, the present study was carried out (1) to determine the cardiorespiratory reflex responses to intravenous injection of 5-HT in spontaneously breathing rats; (2) to evaluate the role of CSLV afferents in eliciting these responses by selectively blocking the conduction of these afferents in spontaneously breathing rats; (3) to determine the stimulatory effect of 5-HT on individual CSLV afferent by using the single-fiber recording technique in artificially ventilated rats; (4) to study whether this stimulatory effect of 5-HT is generated by a direct action on these CSLV neurons using calcium image techniques in isolated vagal pulmonary sensory neurons; and (5) to determine the contribution of 5-HT_3_ receptors to these 5-HT-induced responses by pretreatment with tropisetron, a selective antagonist of 5-HT_3_ receptors, in these neurons.

## Materials and Methods

This study consisted of both *in vivo* and *in vitro* experiments. The experimental procedures described below were in accordance with the recommendation in Guide for the Care and Use of Laboratory Animals published by the National Institutes of Health, and also approved by Institutional Animal Care Use Committee of University of Kentucky and Taipei Medical University.

### *In vivo* Study

#### Animal Preparation

Male Sprague-Dawley (SD) rats (body weight: 310–435 g) were initially anesthetized with an intraperitoneal injection of α-chloralose (100 mg/kg) and urethane (500 mg/kg) dissolved in a 2% borax solution. During the experiment, supplemental doses of these anesthetics were administrated intravenously to maintain the elimination of pain reflexes produced by pinching the rat’s tail. The left jugular vein was cannulated for the injections of anesthetics and pharmacological agents. The right femoral artery was cannulated to measure the arterial blood pressure (ABP). Throughout the experiment, body temperature was maintained at ∼36°C by using a servo-controlled heating pad. At the end of the experiment, the animals were euthanized using an intravenous injection of KCl.

#### Measurement of Cardiorespiratory Reflex Responses

Rats breathed spontaneously via a tracheal cannula. Respiratory flow was measured with a pneumotachograph coupled with a differential pressure transducer (Validyne MP45-14, Northridge, CA, United States) and was integrated to give tidal volume (*V*_T_). All physiological signals were recorded on a chart recorder (Gould RS3200, Cleveland, OH, United States) and a tape recorder (Neuro Data Neurocorder DR-890, New York, NY, United States) for later analysis. Heart rate (HR), ABP, expiratory durations (*T*_E_), and *V*_T_, were analyzed on a breath-by-breath basis. These measurements were made continuously for at least 10 breaths before (baseline) and 30 breaths after the injection. The lungs were hyperinflated (*P*_t_ > 20 cm H_2_O) to establish a constant volume history 3 min before each injection.

#### Measurement of Activity of CSLV Afferents

Activity of single-unit lung afferents was recorded from the right vagus nerve using the techniques described in our previous studies (Hsu et al., 2013). Briefly, the animals were anesthetized, and ventilated by a respirator (Harvard 683, South Natick, MA, United States) via a tracheal cannula inserted just below the larynx. The right cervical vagus nerve was separated and placed on a small dissecting platform. A thin filament was teased away from the desheathed nerve trunk and placed on a platinum-iridium hook electrode. The thin filament was split until the afferent activity arising from a single unit was electrically isolated. Only afferent fibers that met the following criteria were studied in this present study: (1) fibers with a short latency (<2 s) and intense response to the capsaicin injection (1.5 μg/kg), and (2) fibers where the general locations of the receptors could be identified by their responses to the gentle pressing of the lungs with a glass rod at the end of the experiment. The fiber activity of CSLV afferents, tracheal pressure (*P*_t_) and ABP were recorded on a thermal writer (Gould TA11, Cleveland, OH, United States) and on a VCR format data recorder (Neurocorder DR-890). Fiber activity (FA) was analyzed by a computer in 0.5 s intervals. Increase in FA (ΔFA) was calculated as the difference between peak FA and baseline FA. A C-fiber was considered to be activated when ΔFA exceeded 0.5 impulses/s. Because the receptor field of the CSLV fibers needed to be identified by pressing the lungs with a glass rod in an open-chest preparation. Responses of breathing pattern and afferent activity were not measured in the same group of rats.

#### Experimental Protocol

Five series of experiments were carried out.

##### Study series 1

The ventilatory and cardiovascular responses elicited by intravenous bolus injections of increasing doses of 5-HT (4, 8, and 16 μg/kg) were determined in 6 rats; these injections was performed in alternative order to achieve a balanced design. To avoid any accumulated effect, at least 15 min elapsed between two injections in this and subsequent protocols.

##### Study series 2

To study the role of CSLV afferents, 5-HT-induced cardiorespiratory responses was determined before and after a bilateral perineural capsaicin treatment (PCT), which produces a selective and reversible blockade of the C-fiber conduction in the vagus nerves (Jancso and Such, 1983; Lee et al., 1996). The method for PCT was previously described (Lin and Lee, 2002). Briefly, cotton strips soaked in capsaicin solution (300 μg/ml) were wrapped around a 2–3 mm segment of the isolated cervical vagi for 20 min and then removed. To verify the effectiveness and specificity of the blocking effect of PCT on the vagal C fibers under the present experimental conditions, our criterion for a successful PCT was judged on the abolition of the reflex responses induced by the injection of capsaicin (1 μg/kg) and no influence on lung inflation (*P*_t_ = 20 cm H_2_O) triggers apnea that is known to result from stimulation of pulmonary stretch receptors (Coleridge and Coleridge, 1984). At the end, 5-HT injection was repeated after bilateral cervical vagotomy.

##### Study series 3

To determine the role of the 5-HT_3_ receptor, 5-HT-induced cardiorespiratory response were studied before and 15 min after either a pretreatment of tropisetron (15 μg/kg; *n* = 6), a selective antagonist of 5-HT_3_ receptor, or its vehicle (*n* = 6).

##### Study series 4

The CSLV afferent responses elicited by intravenous bolus injections of increasing doses of 5-HT (4, 8, and 16 μg/kg) were determined in 10 CSLV fibers; these injections was performed in alternative order to achieve a balanced design.

##### Study series 5

To determine the role of the 5-HT_3_ receptor, 5-HT-induced afferent responses were studied before and 15 min after either a pretreatment of tropisetron (15 μg/kg; *n* = 8), a selective antagonist of 5-HT_3_ receptor, or its vehicle (*n* = 6). To further determine the specificity of the antagonistic effect of tropisetron on 5-HT_3_ receptors, three other selective stimulants of lung C fibers, capsaicin (0.8 μg/kg; *n* = 15), PBG (4–8 μg/kg; *n* = 12) and lactic acid (9 or 18 mg/kg; *n* = 11), were chosen for this study, following the same protocol.

### *In vitro* Study

#### Identification of Lung Vagal Sensory Neurons

Lung vagal sensory neurons were identified by retrograde labeling with the fluorescent tracer, 1,1′-dioctadecyl-3,3,3′,3′-tetramethylindocarbocyanine perchlorate (DiI), following a protocol as described previously (Kwong and Lee, 2002; Hsu and Lee, 2015). In brief, young male SD rats (40–120 g) were anesthetized by aerosolized isoflurane (2% in O_2_) through a nose cone connected to a vaporizing machine (AM Bickford, New York City, NY, United States). A small midline incision was made on the ventral neck skin. The DiI (0.2 mg/ml; 1% ethanol concentration; 0.05 ml) was instilled into the lungs through a needle (30 gauge) inserted into the trachea lumen, and the incision was then closed. After 7–11 days instillation, the animals were euthanized for the cell culture.

#### Isolation of Nodose and Jugular Ganglion Neurons

The methodology was described in detail in our previous studies (Kwong and Lee, 2002; Hsu and Lee, 2015). The DiI-labeled rats were anesthetized with 5% isoflurane inhalation and decapitated. The head was immersed in an ice-cold DMEM/F-12 solution, followed by the extraction of the nodose and jugular ganglia. Each ganglion was desheathed, cut, placed into a mixture of type IV collagenase (0.04%) and dispase II (0.02%), and incubated for 60 min in 5% CO_2_ in air at 37°C. The ganglion suspension was centrifuged (150 *g*, 5 min), and the supernatant was aspirated. The pellet was then resuspended in a modified DMEM/F12 solution and gently triturated. The dispersed cell suspension was centrifuged (500 *g*, 8 min) through a layer of bovine serum albumin (15%) to separate the cells from the myelin debris. The pellets were resuspended in the modified DMEM/F12 solution plated onto poly-L-lysine-coated glass coverslips and were then incubated overnight (5% CO_2_ in air at 37°C). Cells isolated from nodose and jugular ganglia were cultured and studied separately in each experiment. However, data obtained from these two ganglion neurons were pooled for analysis because no noticeable difference was found between their responses to 5-HT.

#### Measurement of Ca^2+^ Transients

Ca^2+^ transients were measured in CSLV neurons with a Zeiss digital fluorescence microscope and a digital CCD camera (Princeton Instruments, Trenton, NJ, United States), as previously described (Gu et al., 2007; Hu et al., 2010; Hsu et al., 2013). Briefly, intracellular Ca^2+^ was monitored using the fluorescent Ca^2+^ indicator fura-2 AM. Neurons were loaded with 5 μM fura 2-AM (Molecular Probes, Eugene, OR, United States) for 30 min at 37°C in tissue culture medium and then rinsed with ECS and allowed to deesterify for at least 30 min before use. Dual images (340- and 380-nm excitation, 510-nm emission) were collected, and pseudocolored ratiometric images were monitored during the experiments. The coverslip containing cells was then mounted into a chamber (0.2 ml) placed on the stage of the fluorescence inverted microscope. The recording chamber was perfused continuously with ECS or the test chemicals by a gravity-fed valve control system (VC-66CS; Warner Instruments, Hamden, CT, United States). The intracellular Ca^2+^ concentration ([Ca^2+^]_i_) was continually analyzed at 2-s intervals during the experiments by using the Axon Imaging Workbench software (Axon Instruments, Union City, CA, United States). An increase in [Ca^2+^]_i_ (Δ[Ca^2+^]_i_) was measured as the difference between the peak amplitude of Ca^2+^ transients (4-s average) and the 30-s average at baseline. A neuron was considered to be activated when Δ[Ca^2+^]_i_ exceeded 10 nM.

#### Experimental Protocols

The CSLV neurons were selected from the cultured cells for analysis that met the following criteria: (1) a spherical shape with no neurite outgrowths, (2) activated by capsaicin (1 μM, 30 s), (3) labeled with DiI fluorescence. Two study series were performed:

##### Study series 1

Study series 1 was performed to determine the effect of three concentrations of 5-HT (0.3, 1, and 3 μM; 30 s) on the responses of Ca^2+^ transients in CSLV neurons. Fifteen min elapsed between two applications for recovery. In the end of the experiment, a KCl solution (60 mM; 15 s) was applied to test the cell vitality.

##### Study series 2

Study series 2 was performed to evaluate the role of 5-HT_3_ receptors: the 5-HT (3 μM; 30 s) evoked Ca^2+^ transients were compared before and after the pretreatment of tropisetron (10 nM; 5 min).

#### Chemicals

All chemicals were purchased from Sigma-Aldrich (St. Louis, MO, United States). Stock solutions of 5-HT (2.5 mg/ml), tropisetron (1 mg/ml) and PBG (400 μg/ml) were prepared in isotonic saline. A stock solution of capsaicin (250 μg/ml) was prepared in 1% ethanol, 1% Tween 80, and 98% saline. Solutions of these chemicals at desired concentrations were then prepared daily by dilution with saline (*in vivo* study) or ECS (*in vitro* study). The effective doses and treatment protocols for these drugs were adopted from our previous studies (Hong et al., 1997; Gu and Lee, 2006; Lee and Yu, 2014) or determined in our preliminary study. The adequacy of doses and selectivity of 5-HT_3_ receptor antagonists were verified by the results that the selected doses of these drugs could completely block the responses to their specific receptor agonists but did not affect the responses to capsaicin.

#### Data Analysis

Data were analyzed with paired *t*-test, one-way or two-way repeated-measures ANOVA tests followed by a *post hoc* Fisher’s least significant difference test. A *P*-value < 0.05 was considered significant. All data are means ± SEM.

## Results

### *In vivo* Study

The *in vivo* study was carried out in 47 rats (345 ± 5 g). Among these 47 rats, a total of 24 rats were used in the study series 1–3; a total of 25 lung C-fiber afferents were studied in 23 rats of study series 4 and 5.

#### Study Series 1

**FIGURE 1:**
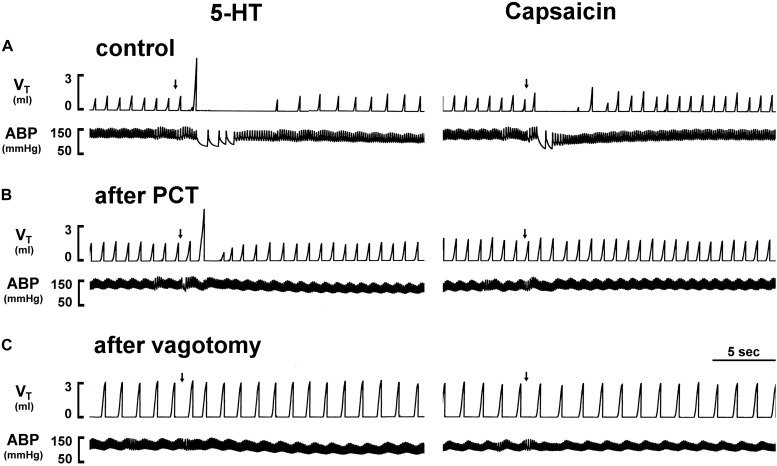
Experimental records illustrating the effect of perineural capsaicin treatment (PCT) and vagotomy of both cervical vagi on cardiorespiratory responses to injections of 5-HT and capsaicin in an anesthetized rat (body weight: 320 g). Left panels: responses to right-atrial injections of 5-HT (8 μg/kg) before (as control) **(A)**, 15 min after PCT **(B)**, and 20 min after vagotomy **(C)**. Right panels: responses to right-atrial injections of capsaicin (0.75 μg/kg) before (as control) **(A)**, 15 min after PCT **(B)** and 20 min after vagotomy **(C)**. Injection (0.1 ml) was first slowly injected into the catheter (dead space, 0.2 ml) and then flushed (at arrow) into the circulation by a bolus with saline (0.3 ml). *V*_T_, tidal volume; ABP, arterial blood pressure.

**FIGURE 2:**
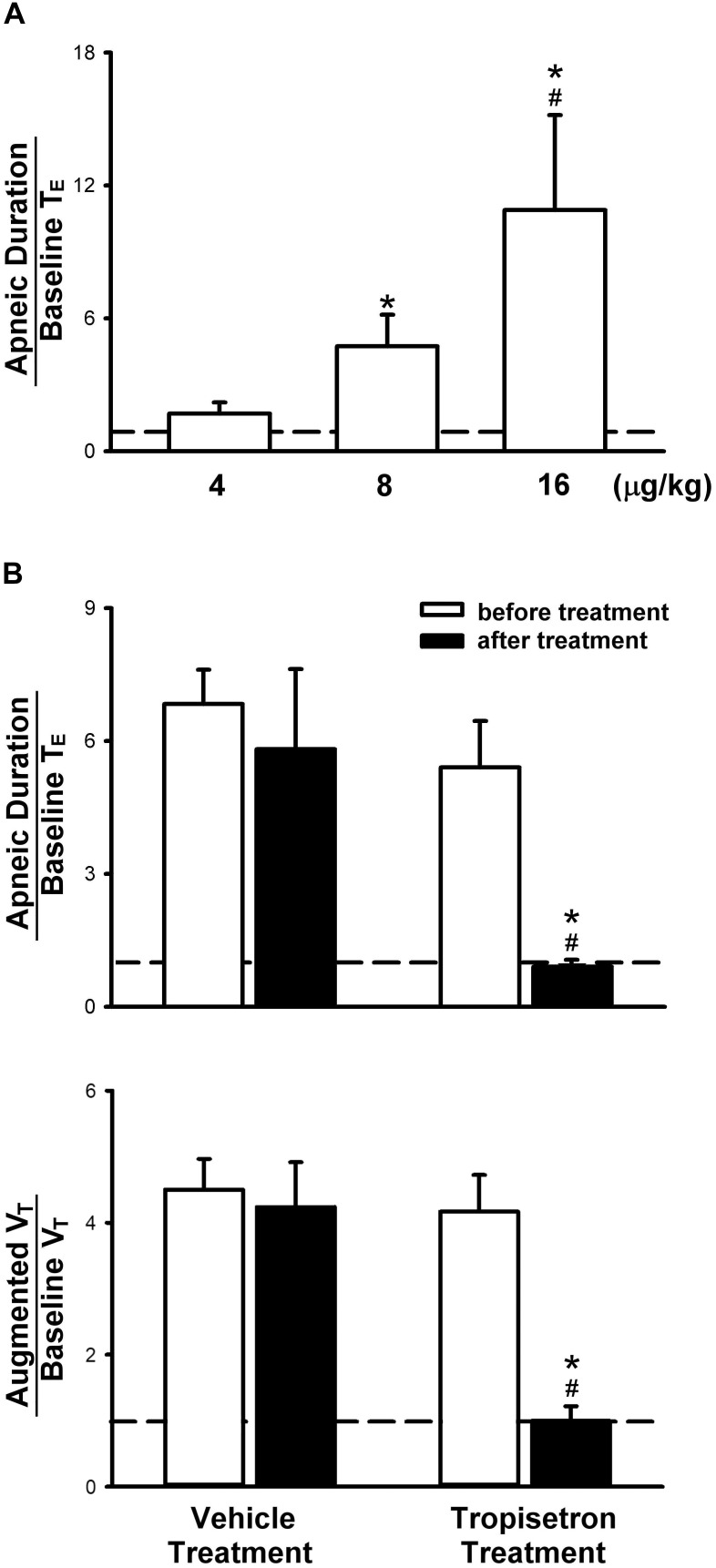
Apneic responses to intravenous injections of 5-HT in anesthetized, spontaneously breathing rats. **(A)** Effect of increasing doses of 5-HT on apneic responses in a group of 6 rats. Symbols ^∗^ and # depict significantly different (*P* < 0.05) from the responses to 4 and 8 μg/kg of 5-HT, respectively. **(B)** Apneic (upper panel) and augmented breath (lower panel) responses to 5-HT (8 μg/kg) before and after pretreatment of tropisetron (15 μg/kg) or its vehicle (saline) in two groups of rats (6 for each group). Data are mean ± SEM. One-way and two-way ANOVA tests followed by a *post hoc* Fisher’s least significant difference test for **(A,B)**, respectively. ^∗^, significantly different from the corresponding responses before tropisetron (*P* < 0.05); #, significant difference when corresponding data between vehicle and tropisetron were compared (*P* < 0.05).

In anesthetized, vagi-intact rats, an intravenous bolus injection of 5-HT elicited an immediate apnea and augmented breath; the latter was characterized by a single exceedingly large inhaled tidal volume (e.g., Figure 1), which were accompanied by initial hypotension and bradycardia, and followed by a more sustained pressor response (e.g., Figure 1 and Table 1). The apneic responses to 5-HT injections were dose-dependent (Figure 2A). The apneic ratio evoked by the high dose 5-HT (16 μg/kg) was 10.89 ± 4.29, which was significantly different from the response by the moderate dose 5-HT (8 μg/kg; apneic ratio: 4.74 ± 1.42, *P* < 0.05) and the low dose 5-HT (4 μg/kg; apneic ratio: 1.70 ± 0.50, *P* < 0.05, *n* = 6) (Figure 2A).

**Table 1:** Cardiovascular responses to intravenous bolus injections of three different doses of 5-HT in a group of spontaneously breathing rats.

	Initial responses		Delayed responses
5-HT injections (μg/kg)	% decrease in ABP	% decrease in HR	% increase in ABP
4	4.3 ± 1.5	7.8 ± 2.2	5.8 ± 1.1
8	34.3 ± 3.3^∗^	45.8 ± 2.1^∗^	12.8 ± 3.4^∗^
16	46.2 ± 3.2^∗#^	54.0 ± 3.8^∗^	21.2 ± 1.6^∗#^

*ABP, arterial blood pressure; HR, heart rate. % decrease in ABP or HR = [(baseline data − mean peak reduction data)/baseline data] × 100%. % increase in ABP = [(mean peak elevation data - baseline data)/baseline data] × 100%. The baselines are values averaged over 10-s periods immediately before 5-HT injections. The mean peak responses are the lowest or highest values averaged over 2-s periods during the first 30 s after injections. Data are mean ± SEM of 6 rats. One-way ANOVA test followed by a post hoc Fisher’s least significant difference test. ^∗^ and #, significantly different (P < 0.05) from the responses to 4 and 8 μg/kg 5-HT, respectively.*

#### Study Series 2

**FIGURE 3:**
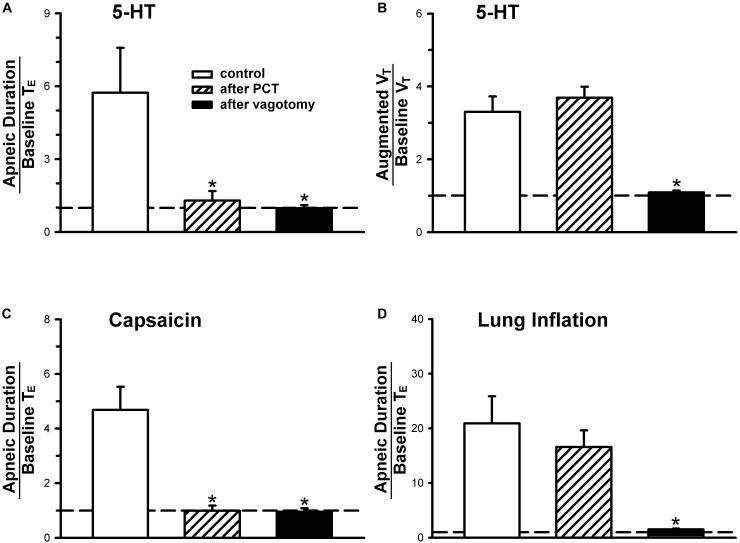
**(A,C,D)** Effect of perineural capsaicin treatment (PCT) and vagotomy on apneic responses to right-atrial injections of 5-HT, capsaicin (0.8 μg/kg) and lung inflation, respectively. **(B)** Effect of PCT and vagotomy on augmented breath responses to right-atrial injections of 5-HT. Data are mean ± SEM. One-way ANOVA test followed by a *post hoc* Fisher’s least significant difference test. ^∗^, significantly different from the control responses (*P* < 0.05).

An intravenous bolus injection of capsaicin (0.8 μg/kg) evoked an immediate apnea, accompanied by hypotension and bradycardia in anesthetized, vagi-intact rats (e.g., Figure 1). These cardiorespiratory responses (apnea, bradycardia, and hypotension) elicited by both 5-HT (8 μg/kg) and capsaicin were similarly abolished by the PCT of vagi (Figure 1, 3): the apneic ratio evoked by 5-HT was 5.73 ± 1.86 before PCT, and 1.29 ± 0.39 after PCT (*P* < 0.05, *n* = 6) (Figure 3A); the apneic ratio evoked by capsaicin was 4.69 ± 0.84 before PCT, and 0.99 ± 0.19 after PCT (*P* < 0.05, *n* = 6) (Figure 3C). However, the lung inflation-induced apnea did not alter by PCT (apneic ratio before PCT: 20.89 ± 4.98, after PCT: 16.57 ± 3.07; *P* > 0.05, *n* = 6) (Figure 3D). Furthermore, augmented breaths were consistently elicited by 5-HT even after PCT (Figure 1A,B: left panel, 3B). After PCT, the initial hypotension and bradycardia to 5-HT injection were nearly completely abolished, whereas the delayed pressor response persisted (Table 2).

**Table 2:** Effect of PCT, tropisetron, and its vehicle (saline) on cardiovascular responses to intravenous bolus injections of 5-HT in three groups of spontaneously breathing rats.

	Initial responses		Delayed responses
	% decrease in ABP	% decrease in HR	% increase in ABP
Control	40.2 ± 3.8	45.8 ± 4.2	10.0 ± 1.8
After PCT	7.0 ± 1.3^∗^	3.8 ± 0.9^∗^	10.5 ± 1.7
Control	28.0 ± 3.7	47.7 ± 6.9	11.5 ± 2.1
After tropisetron	4.0 ± 1.0^∗^	5.7 ± 1.4^∗^	14.2 ± 2.9
Control	34.3 ± 3.7	41.2 ± 3.4	12.2 ± 2.3
After vehicle	27.5 ± 2.7	45.8 ± 4.6	15.0 ± 1.9

*Control, response before treatment. PCT, bilateral perineural capsaicin treatment. Data are mean ± SEM of 6 rats for each group. Paired t-test. ^∗^, significantly different (P < 0.05) from its corresponding control responses. See legend of Table 1 for further explanation.*

#### Study Series 3

Pretreatment of tropisetron (15 μg/kg) completely abolished the 5-HT (8 μg/kg)-induced apnea, the apneic ratios were 5.41 ± 1.05 and 0.90 ± 0.15 before and after tropisetron pretreatment, respectively (*P* < 0.05, *n* = 6) (Figure 2B). And, the response after tropisetron pretreatment was distinctly smaller than the response with pretreatment of vehicle of tropisetron (5.82 ± 1.80, *P* < 0.05, *n* = 6) (Figure 2B, upper panel). In addition, the augmented breath responses to 5-HT were also totally abolished by tropisetron pretreatment (*P* < 0.05, *n* = 6) (Figure 2B, lower panel). After pretreatment of tropisetron, the initial responses of hypotension and bradycardia to 5-HT injection were almost eliminated, whereas the delayed pressor response was not influenced. Both initial and delayed cardiovascular responses to 5-HT injection were not affected by pretreatment with vehicle of tropisetron (Table 2).

#### Study Series 4

**FIGURE 4:**
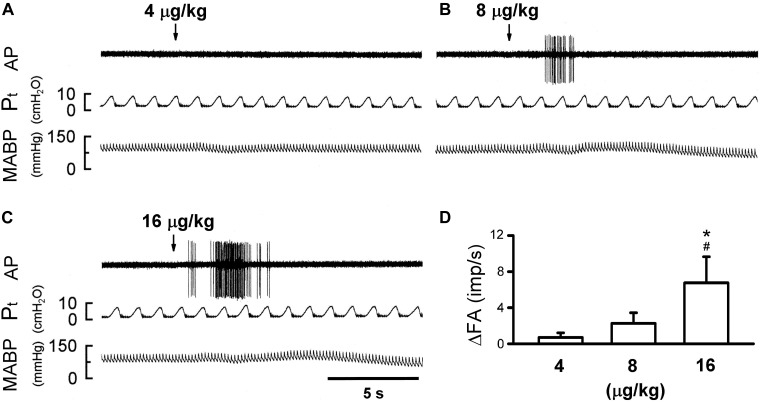
Effect of increasing doses of 5-HT on activity of capsaicin-sensitive lung vagal (CSLV) afferents in anesthetized, open-chest and ventilated rats. **(A–C)** Experimental records illustrating responses of a CSLV afferent to right-atrial injections (arrows) of 4, 8, and 16 μg/kg 5-HT in an anesthetized and open-chest rat (body weight: 405 g), respectively. ΔFA, difference between peak FA (5-s average) occurring within 20 s after the injection and baseline FA (10-s average). AP, action potential; *P*_t_, tracheal pressure; ABP, arterial blood pressure. **(D)** Effect of increasing doses of 5-HT on afferent responses of 10 CSLV fibers in 8 rats. Data are mean ± SEM. One-way ANOVA test followed by a *post hoc* Fisher’s least significant difference test. ^∗^ and #, significantly different (*P* < 0.05) from the responses to 4 and 8 μg/kg of 5-HT, respectively.

The bolus injection of the highest dose of 5-HT (16 μg/kg) activated 80% (8/10) of the C fibers tested; most of them started to discharge within 2 s, and the firing activity reached the peak in ∼5 s after the injection (Figure 4C). 5-HT stimulated lung C-fiber afferents in a dose-dependent manner (e.g., Figure 4), the ΔFA triggered by three different doses of 5-HT were as follows: 0.70 ± 0.51, 2.27 ± 1.16, and 6.76 ± 2.88 impulses/s in 4, 8, and 16 μg/kg of 5-HT group (Figure 4D). The *P*_t_ slightly but significantly elevated after 5-HT injection, the peak *P*_t_ during tidal breathing increased from 8.44 ± 0.25 cm H_2_O (baseline) to 10.23 ± 0.32 cm H_2_O (*P* < 0.05, *n* = 10) after the injection of highest dose of 5-HT (Table 3). These increases in *P*_t_ gradually returned toward the baseline after 5–10 min.

**Table 3:** Tracheal pressure (*P*_t_) before (baselines) and after intravenous bolus injections of 5-HT under various experimental conditions in two groups of anesthetized, artificially ventilated rats.

	Baselines (cm H_2_O)	After 5-HT injections (cm H_2_O)
**(A) 5-HT injections**		
4 μg/kg	8.48 ± 0.31	8.73 ± 0.14
8 μg/kg	8.61 ± 0.11	9.54 ± 0.27^∗#^
16 μg/kg	8.44 ± 0.25	10.23 ± 0.32^∗#^
**(B) 5-HT injections (16 μg/kg)**		
Before tropisetron	9.47 ± 0.16	10.31 ± 0.71
After tropisetron	8.68 ± 0.25	9.89 ± 0.13^∗^

*(A) Tracheal pressure (P_t_) response to injections of 3 different doses of 5-HT in a group of 10 rats. (B) P_t_ response to injections of 5-HT (16 μg/kg) before and after tropisetron pretreatment in a group of 8 rats. The baselines were values averaged over 10-s periods immediately before 5-HT injections. The peak responses are the highest values averaged over 2-s periods during the first 30 s after injections. Data are mean ± SEM. One-way ANOVA test followed by a post hoc Fisher’s least significant difference test. ^∗^, significantly different (P < 0.05) from its corresponding baseline responses. #, significantly different (P < 0.05) from the responses to 4 μg/kg of 5-HT.*

#### Study Series 5

**FIGURE 5:**
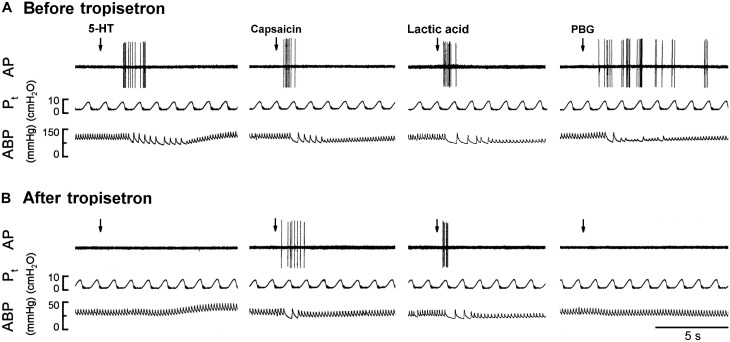
Experimental records illustrating the responses of a capsaicin-sensitive lung vagal afferent arising from the right upper lobe to 5-HT (16 μg/kg), capsaicin (0.8 μg/kg), lactic acid (18 mg/kg), or phenylbiguanide (PBG; 6 μg/kg) before and after pretreatment with tropisetron in an anesthetized, open-chest and ventilated rat (body weight: 410 g). **(A)** Responses before tropisetron pretreatment (15 μg/kg). **(B)** Responses to 5-HT, capsaicin, lactic acid and PBG at 15 min after tropisetron pretreatment, respectively. Stimulant injections were marked by the arrows. AP, action potential; *P*_t_, tracheal pressure; ABP, arterial blood pressure.

**FIGURE 6:**
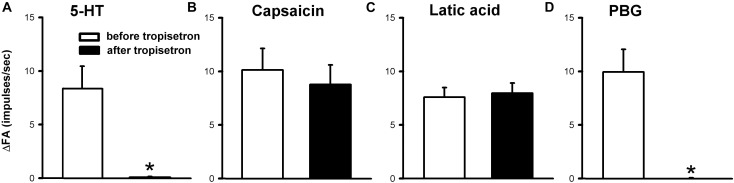
Effect of pretreatment with tropisetron (15 μg/kg) on capsaicin sensitive lung vagal afferent responses to various stimulants in anesthetized, open-chest rats. The afferent responses to right-atrial injections of 5-HT (16 μg/kg, *n* = 15; **A**), capsaicin (0.8 μg/kg, *n* = 15; **B**), lactic acid (9 or 18 mg/kg, *n* = 11; **C**) and phenylbiguanide (PBG, 4–8 μg/kg, *n* = 12; **D**). ΔFA, difference between peak FA (2-s average for capsaicin and lactic acid; 5-s average for 5-HT and PBG) occurring within 20 s after the injection and baseline FA (10-s average). Data are mean ± SEM. Paired *t*-test ^∗^, significantly different (*P* < 0.05) from the corresponding responses before tropisetron.

The 5-HT-induced activation of lung C-fiber afferents was totally abolished by the tropisetron pretreatment (e.g., Figure 5); after the tropisetron pretreatment, the ΔFA induced by 5-HT was 0.09 ± 0.07 impulses/s, which was significantly different from the ΔFA induced by 5-HT before tropisetron pretreatment (8.36 ± 2.10 impulses/s; *P* < 0.05, *n* = 15) (Figure 6A). Pretreatment of tropisetron also completely abolished the activation of lung C-fiber afferents induced by PBG (4–8 μg/kg; ΔFA before and after tropisetron: 9.96 ± 2.10 and 0.04 ± 0.03 impulses/s, *P* < 0.05, *n* = 12), but did not affect that induced by capsaicin (0.8 μg/kg; *P* > 0.05, *n* = 15) and lactic acid (9 or 18 mg/kg; *P* > 0.05, *n* = 11) (Figure 5, 6). After the pretreatment with tropisetron, the injection of 5-HT (16 μg/kg) still caused a significant increase in *P*_t_ (Table 3).

### *In vitro* Study

The Ca^2+^-transient experiments were performed in 29 CSLV neurons isolated from nodose and jugular ganglia of 12 rats (87 ± 4 g) and identified by the fluorescent tracer of DiI. The average baseline [Ca^2+^]_i_ was 79.56 ± 6.62 nM.

#### Study Series 1

**FIGURE 7:**
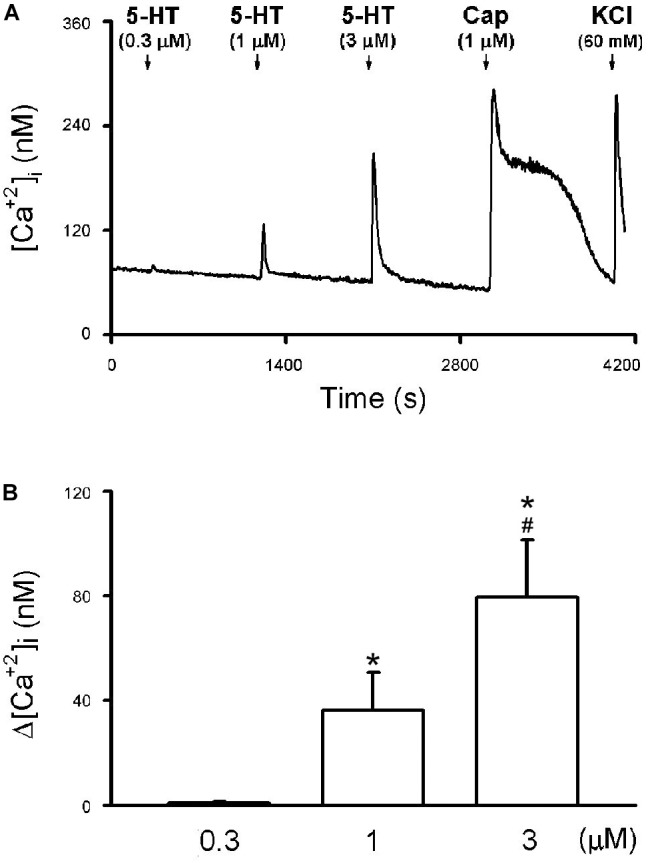
5-HT-evoked calcium transients in isolated rat capsaicin-sensitive lung vagal (CSLV) neurons. **(A)** An experimental record illustrating that 5-HT concentration-dependently evoked calcium transients in a jugular ganglion neuron (diameter: 28 μm). 5-HT and capsaicin (Cap; 1 μM) was applied for 30 s each, and potassium chloride (KCl; 60 mM, 15 s) was applied to test cell viability at the end of experiments. Time between 2 consecutive 5-HT challenges was 15 min. [Ca^2+^]_i_, intracellular concentration of Ca^2+^. **(B)** Group data of 5-HT evoked increase of [Ca^2+^]_i_ (Δ[Ca^2+^]_i_) obtained from 21 CSLV neurons in 12 rats. Data are mean ± SEM. One-way ANOVA test followed by a *post hoc* Fisher’s least significant difference test. Symbols ^∗^ and # depict significantly different (*P* < 0.05) from the responses to 0.3 and 1 μM of 5-HT, respectively.

The application of 5-HT (30 s) evoked a rapid and transient increase in the [Ca^2+^]_i_ (Δ[Ca^2+^]_i_) in a concentration-dependent manner in CSLV neurons (e.g., Figure 7). The Δ[Ca^2+^]_i_ evoked by 0.3, 1, and 3 μM of 5-HT was 0.78 ± 0.32, 36.35 ± 13.97, and 79.73 ± 22.10 nM, respectively (*P* < 0.05, *n* = 21) (Figure 7B). The neuronal sensitivity to capsaicin (1 μM, 30 s) was demonstrated by a significant increase in [Ca^2+^]_i_ (Δ[Ca^2+^]_i_ = 82.90 ± 9.02 nM, *n* = 21) verifying these cells as CSLV neurons. At the end of the experimental run, a KCl solution (60 mM; 15 s) was applied to test cell vitality (e.g., Figure 7A).

#### Study Series 2

**FIGURE 8:**
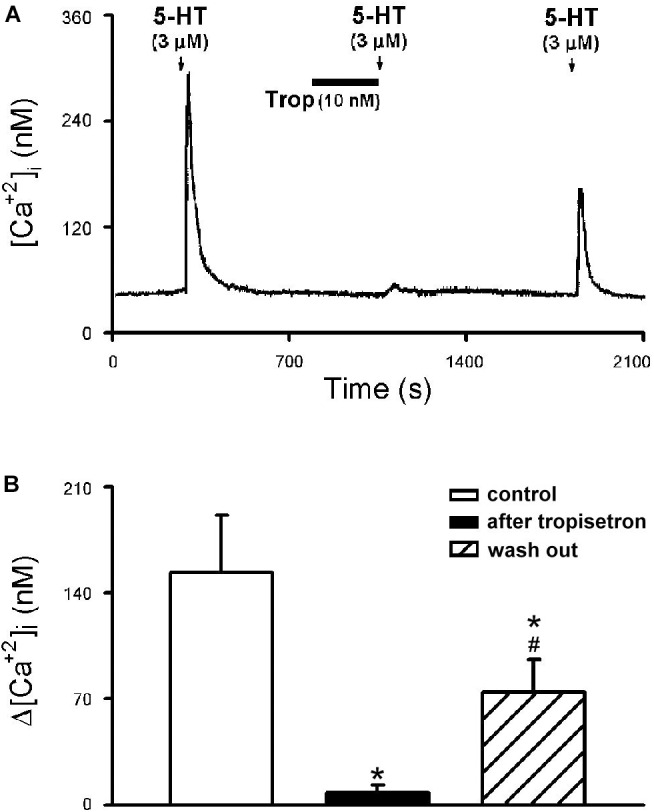
Effect of tropisetron on 5-HT-induced calcium transients in isolated rat capsaicin-sensitive lung vagal (CSLV) neurons. **(A)** An experimental record illustrating that pretreatment with tropisetron (Trop; 10 nM, 5 min) almost completely prevented the Ca^2+^ transient evoked by 5-HT (3 μM, 30 s) in a nodose ganglion neuron (diameter: 24 μm). **(B)** Group data of 5-HT evoked increase of [Ca^2+^]_i_ (Δ[Ca^2+^]_i_) before and after tropisetron pretreatment obtained from 8 CSLV neurons in 5 rats. Time between 2 consecutive 5-HT challenges was 15 min. Data are mean ± SEM. One-way ANOVA test followed by a *post hoc* Fisher’s least significant difference test. ^∗^ and #, significantly different (*P* < 0.05) from the responses before and immediately after tropisetron, respectively.

The 5-HT (3 μM)-induced Ca^2+^ transient was nearly completely abolished by pretreatment of tropisetron (10 nM; 5 min) (Figure 8A); the Δ[Ca^2+^]_i_ before and after tropisetron pretreatment were 153.38 ± 37.83 and 8.06 ± 5.13 nM, respectively (*P* < 0.05, *n* = 8) (Figure 8B). The 5-HT induced Ca^2+^ transient partially recovered after a 20-min washout of tropisetron application (74.30 ± 21.81 nM, *P* < 0.05, *n* = 8) (Figure 8B).

## Discussion

The present study demonstrated that, in anesthetized spontaneous breathing rats, intravenous bolus injection of 5-HT (8 μg/kg) immediately evoked an augmented inspiration and/or apnea, hypotension and bradycardia. These initial responses were then followed by a more sustained pressor response. The apnea accompanied by hypotension and bradycardia is a typical triad of pulmonary chemoreflex responses resulting from CSLV-afferent stimulation (Lee and Pisarri, 2001; Lee and Yu, 2014). Indeed, they were completely eliminated after selective blockade of the conduction of vagal C fibers with the PCT (Jancso and Such, 1983; Lee et al., 1996; Lin and Lee, 2002), suggesting that the stimulation of CSLV afferents is primarily responsible for triggering these 5-HT-induced responses. In anesthetized and artificially ventilated rats, our electrophysiological experiments have further demonstrated that 5-HT stimulated the individual CSLV afferents in a dose-dependent manner. In addition, the isolated rat CSLV neurons, 5-HT also evoked Ca^2+^ transients in a concentration-dependent manner, which suggests a direct action of 5-HT on these neurons. These results further argue against a secondary stimulatory effect caused by a possible release of other inflammatory mediators and C-fiber activators from airway tissues or a possible mechanical stimulation resulting from airway constriction that is known to be generated by 5-HT (Szarek et al., 1995).

In addition to 5-HT_3_ receptors, it had been shown that 5-HT_1_ or 5-HT_4_ receptor mRNA were detected in mice lung jugular neurons (Potenzieri et al., 2012). Moreover, 5-HT induced-activation of the mice lung vagal fibers was not influenced by antagonist of 5-HT_3_ (Potenzieri et al., 2012), suggesting an involvement of non-5-HT_3_ mechanism. However, in this study, the stimulatory effect of 5-HT the CSLV-mediated cardiorespiratory reflexes and the electrophysiological responses of CSLV afferents, and the 5-HT-induced elevation of Ca^2+^ transients in isolated neurons were all abolished by the pretreatment with tropisetron, clearly indicating that this stimulating effect is mediated through an action on the 5-HT_3_ receptors expressed in the nerve endings of CSLV afferents. The specificity of the same dose of tropisetron as the antagonist of 5-HT_3_ receptor was further demonstrated by the absence of any blocking effect on the responses to other potent activators of CSLV afferents such as capsaicin (an agonist of transient receptor potential vanilloid 1 receptor; TRPV1) (Hong et al., 1997) and lactic acid, an activator of both acid-sensing ion channel and TRPV1 (Gu and Lee, 2006; Lee and Yu, 2014). Taken together, these results strongly suggest that activation of 5-HT_3_ is responsible for the stimulating action of 5-HT on CSLV neurons, although additional evidence obtained from immunohistochemistry and/or molecular cloning will be required to further confirm the expression in rat lung and airways.

An important role of CSLV in eliciting the pulmonary chemoreflex responses to 5-HT was clearly demonstrated by the responses observed after the PCT treatment. The effectiveness of the PCT treatment was demonstrated by the complete blockade of the apneic reflex evoked by capsaicin, a selective C-fiber activator (e.g., Figure 3C), and the selectivity of the PCT treatment on unmyelinated afferents (including CSLVs) was illustrated by the persistence of apneic reflex induced by lung inflation, which is known to be mediated through activation of pulmonary stretch receptors and conducted by large-diameter myelinated fibers (Coleridge and Coleridge, 1984).

The CSLV fibers recorded in our study are only the ones that did respond to capsaicin, a potent and selective activator to lung vagal C fibers. However, it has been reported that a small fraction Aδ-receptors (conduction velocities = 2.3 ∼ 7.2 m/s) might be stimulated by capsaicin in guinea pig lungs (Adcock et al., 2014). Because of relative difficulty in smaller animals such as the rat, we did not measure the conduction velocities of vagal afferent fibers in the present study. Alternatively, pulmonary C fibers were identified by the criteria established in our previous study (Ho et al., 2001) in which the conduction velocities were determined in the individual rat vagal afferent fibers. Although the majority of pulmonary C-fibers was identified, we cannot rule out the possibility that 5-HT might active lung Aδ-receptors (Zhang et al., 2006).

More importantly, the present study not only demonstrates the critical role of CSLV in eliciting the pulmonary chemoreflex response to 5-HT, but also provides additional information that other types of lung vagal sensory fibers may be also involved in 5-HT-induced airway reflex. A possible role of capsaicin-insensitive pulmonary C-fibers should be considered as Dutta and Deshpande (2010) have reported that PBG, a selective agonist of 5-HT_3_ receptor, activated a subset of pulmonary vagal afferents that did not respond to capsaicin (Dutta and Deshpande, 2010). This possibility was supported by our result that 5-HT-induced augmented breath was unaffected by perineural capsaicin pretreatment (Figure 3B), but was almost completely abolished by tropisetron (Figure 2B, lower panel).

One of the prominent reflex responses elicited by stimulation of RARs is augmented breath (Wang et al., 1996). After the triad of pulmonary chemoreflex responses, apnea, hypotension and bradycardia, were abolished by the PCT treatment that blocked the conduction of unmyelinated C-fibers, the immediate augmented breath elicited by the 5-HT injection continued to persist (Figure 1A,B, 3C) or became even more intense, which was subsequently eliminated by bilateral vagotomy in the same animals (Figure 3B). Thus, these results clearly suggest an involvement of RARs in 5-HT-induced pulmonary reflex responses. RARs, a polymodal receptor, can be activated by mechanical and chemical stimuli (Sant’Ambrogio and Widdicombe, 2001). In our electrophysiological findings that *P*_t_ was elevated after 5-HT injection (Table 3); the increased *P*_t_ caused by decreased lung compliance is a known and effective stimulus of RARs (Pisarri et al., 1990; Widdicombe, 2003). During our electrophysiological recordings, the left vagus nerve was left intact in the rat and, therefore, the 5-HT-induced *P*_t_ elevation may have resulted from a reflex bronchoconstriction via activation of 5-HT_3_ receptors expressed in CSLV fibers. In addition, the bronchoconstriction may be a result of a direct action of 5-HT on airway smooth muscle by activation of 5-HT_2_ receptors (Szarek et al., 1995). This assumption is supported by our observation that the elevation of *P*_t_ induced by 5-HT was not influenced after pretreatment with 5-HT_3_ antagonist (Table 3). In addition to the indirect action of mechanical stimuli, RARs might be stimulated directly by 5-HT via stimulation of 5-HT_3_ receptors. This notion is supported by the fact the augmented breath triggered within 2 breaths after 5-HT injection while changes in lung mechanics might not have sufficient time to develop. Furthermore, that 5-HT-triggered augmented breath is absent after tropisetron pretreatment (Figure 2B, lower panel). The important role of RARs in regulating the overall respiratory functions including breathing pattern, cough and other airway reflexes has been extensively reported in the literature (Lee and Yu, 2014). Although, some other investigators believe that RARs are mechanoreceptors, while the direct chemical stimulation of 5-HT, if any, may come from activation of lung Aδ afferents (Yu, 2002; Lee and Yu, 2014). Whether the 5-HT exerts a stimulatory effect on RARs and the underlying mechanisms of its action remain to be further investigated.

Potenzieri et al. (2012) used an *ex vivo* vagally innervated mouse lung preparation and recorded the extracellular neural activity of C-fibers by inserting the recording electrode directly into nodose and jugular ganglia. Their study showed 5-HT activates a high percentage of CSLV afferents of both nodose (73%) and jugular (80%) origins, but only the responses of nodose afferents were abolished by the 5-HT_3_ receptor antagonist, indicating the different phenotypes of lung afferents related to the ganglion origin (Potenzieri et al., 2012; Mazzone and Undem, 2016). In the present study, C-fiber activities were recorded in rats in the whole animal preparation without differentiating them based on the ganglion origin, and our results showed that 5-HT stimulated a similarly high percentage (∼80%) of CSLV afferents as in their study (Potenzieri et al., 2012). However, our study showed that the 5-HT-induced stimulatory effect on all CSLV afferents was totally eliminated by a pretreatment of 5-HT_3_ antagonist, indicating a lack of difference in the responses from cells of different ganglionic origins. It is possible that when 5-HT was injected intravenously as a bolus in our study, it may have preferentially activated the CSLV afferents innervating the lung, which originated primarily from nodose ganglia, and to a much less extent the C-fibers innervating the conducting airways, which originated from jugular ganglia. This could explain, at least in part, the discrepancy between these two studies because the conducting airways (trachea and bronchi) receive blood supplies from the bronchial circulation (via the left heart). Similar conclusion was also found in our study of Ca^2+^ transient recorded in isolated CSLV neurons; perfusion of 5-HT activated 69.2% (9 in 13) and 62.5% (5 in 8) of the neurons isolated from nodose and jugular ganglia, respectively, but the 5-HT-evoked activation of CSLV neurons from both origins was totally blocked by a pretreatment of 5-HT_3_ antagonist (Figure 8), suggesting a possible species-dependent difference in the role of 5-HT_3_ receptor in mediating the stimulatory effect of 5-HT on vagal pulmonary sensory neurons.

Hypotension and bradycardia, accompanying the apnea and augmented breath, were immediately elicited by the intravenous injection of 5-HT (Figure 1 and Table 1) and abolished by pretreatment of either tropisetron or PCT, indicating that the stimulation of the 5-HT_3_ receptors expressed in the CSLV afferents was probably involved (Table 2). Although we cannot completely rule out the possibility that the 5-HT_3_ receptors located on the vagal sensory nerves in the heart may have also contributed to these responses (Villalon and Centurion, 2007), the short latency (approximately 1–2 s) of the onset of these responses after the bolus intravenous injection of 5-HT seem to suggest a more dominant role of CSLV fibers in eliciting these cardiovascular responses. The delayed vasopressor response after the 5-HT injection was not affected by either tropisetron or PCT pretreatment (Table 2), which was probably caused by the peripheral vasoconstriction resulting from an activation of 5-HT_2_ receptors located in vascular smooth muscles (Lundberg and Saria, 1987).

The reflex effects generated by activation of CSLV afferents have been extensively documented (Coleridge and Coleridge, 1984; Lee and Pisarri, 2001; Lee and Yu, 2014). Stimulation of these afferents can elicit powerful centrally-mediated reflex responses, including tachypnea, bradycardia, hypotension, bronchoconstriction and airway hypersecretion (Coleridge and Coleridge, 1984; Lee and Pisarri, 2001; Lee and Yu, 2014). Activation of these afferents is also known to cause airway irritation and cough (Coleridge and Coleridge, 1984; Lee and Pisarri, 2001; Lee and Yu, 2014). Activation of these sensory endings triggers the release of these neuropeptides that in turn act on a number of effector cells in the respiratory tract (e.g., airway and vascular smooth muscles, cholinergic ganglia, and inflammatory cells) and elicit local “axon reflex” effects, such as protein extravasation, inflammatory cell chemotaxis, etc. Thus, sustained stimulation of these endings can lead to the development of “neurogenic inflammatory reaction” in the airways (Lundberg and Saria, 1987; De Swert and Joos, 2006). Moreover, these nerve endings become hypersensitive resulting from the release of several mediators due to lung inflammation, a given level of stimulus will then evoke a more sustained and intense respiratory reflexes. Therefore, the sensitization of the C-fiber afferents is probably involved in the pathogenesis of airway inflammatory diseases such as asthma and chronic obstructive pulmonary disease (Barnes, 1992; Nasra and Belvisi, 2009).

5-HT can be released from a variety of cell types in the airways, particularly platelets and mast cells (McNicol and Israels, 1999). An important role of 5-HT in the pathogenesis of allergic airway inflammatory diseases, such as asthma and chronic obstructive pulmonary disease, has been extensively documented (Idzko et al., 2015; Meier et al., 2017). 5-HT plasma level has been shown to correlate positively with clinical status and negatively with pulmonary function in patients with asthma (Lechin et al., 1998). In addition, 5-HT levels are elevated in bronchoalveolar lavage fluid of allergen-sensitized mice and in asthmatic patients after allergen inhalation provocation (Durk et al., 2013). 5-HT is known to exert multiple pathogenic effects in the airway such as bronchoconstriction (Buckner et al., 1993; Martin et al., 1994), increases microvascular permeability (Arreola-Ramirez et al., 2015), and airway edema (Sumita et al., 1989; Lima et al., 2007). Taken together, the involvement of 5-HT in the pathophysiological changes in airway function in allergic airway inflammatory diseases may be mediated, at least in part, through its stimulatory effect on CSLV neurons.

In conclusion, results from the present study have clearly demonstrated a dose-dependent stimulatory effect of 5-HT on CSLV fiber terminals and apnea reflex responses in anesthetized rats. This stimulatory effect was mediated through a direct activation of the 5-HT_3_ receptor expressed in these sensory nerves.

## Ethics Statement

This study was carried out in accordance with the recommendations of Guide for the Care and Use of Laboratory Animals published by the National Institutes of Health, and also approved by Institutional Animal Care Use Committee of University of Kentucky and Taipei Medical University.

## Author Contributions

C-CH, TR, L-YL, and YSL responsible for designing the study, performing experiments, collecting, analyzing, and interpreting the data, and preparation of the manuscript.

## Conflict of Interest Statement

The authors declare that the research was conducted in the absence of any commercial or financial relationships that could be construed as a potential conflict of interest.
